# Ustilaginoidin D Induces Acute Toxicity and Hepatotoxicity in Mice

**DOI:** 10.3390/toxins17050250

**Published:** 2025-05-17

**Authors:** Bo Wang, Xiaolong Bai, Min Zhang, Xiangxiang Liu, Muhammad Zulqar Nain Dara, Lingjing Liu, Mingming Ou, Dayong Li, Jiyang Wang, Ling Liu, Wenxian Sun

**Affiliations:** 1 College of Plant Protection, Jilin Provincial Key Laboratory of Green Management of Crop Pests and Diseases, Jilin Agricultural University, Changchun 130118, China; wangboo@catas.cn (B.W.); xiaolongbai688@gmail.com (X.B.);; 2 College of Plant Protection and the Ministry of Agriculture Key Laboratory of Pest Monitoring and Green Management, China Agricultural University, Beijing 100193, China; 3 Environment and Plant Protection Institute, Chinese Academy of Tropical Agricultural Sciences, Haikou 571101, China; 4 College of Chinese Medicinal Materials, Jilin Agricultural University, Changchun 130118, China; 5 Yunnan Key Laboratory of Cell Metabolism and Diseases, State Key Laboratory for Conservation and Utilization of Bio-Resources in Yunnan, Center for Life Sciences, School of Life Sciences, Yunnan University, Kunming 650091, China

**Keywords:** ustilaginoidin D, rice false smut, mice, hepatotoxicity, human health

## Abstract

Ustilaginoidin D is a type of bis-naphtho-γ-pyrone mycotoxin produced by Ustilaginoidea virens, the causal agent of rice false smut. Although previous studies have demonstrated the inhibitory effect of ustilaginoidin D on ATP synthesis and cancer cell growth in mice, its specific health risks remain unclear. Here, we reveal that ustilaginoidin D is highly toxic to mice with an LD_50_ value of 213 mg /kg·bw. Dose-dependent weight loss and liver damage were observed, accompanied by altered markers of liver cell damage, including the enzyme activities of alanine aminotransferase and aspartate aminotransferase and the content of glutathione in mouse liver. RNA-seq analysis of liver tissues from mice treated with 150 mg of ustilaginoidin D/kg·bw identified significant changes in gene expression profiles, with differentially expressed genes enriched in cancer-related pathways, hypertrophic cardiomyopathy, and metabolic pathways. RT-qPCR data are highly consistent with transcriptome analysis in expression profiles of 22 chemical-carcinogenesis-associated genes. These findings indicate that ustilaginoidin D induces acute toxicity and liver dysfunction in mice, raising serious concerns about its threat to human health.

## 1. Introduction

Rice false smut, a fungal disease caused by the biotrophic fungus *Ustilaginoidea virens*, has emerged as a severe threat to rice production in major rice-growing regions worldwide, leading to substantial economic losses in recent years [1]. *U. virens* colonization of rice florets leads to the formation of characteristic false smut balls generating two major classes of mycotoxins: water-soluble cyclic peptides (ustiloxins) and lipid-soluble bis-naphtho-γ-pyrone derivatives (ustilaginoidins) [2,3,4,5,6,7]. These secondary metabolites not only exhibit diverse biological activities but also pose significant risks to food safety by contaminating rice grains, ultimately threatening both animal and human health.

Among the 27 identified ustilaginoidin derivatives, ustilaginoidin A demonstrates an inhibitory effect on the growth of limb bud cells and brain cells in murine embryos while concurrently suppressing mitochondrial ATP synthesis [8,9,10]. Hemiustilaginoidin F and hemiustilaginoidin D inhibit the radicle and plumule elongation of rice and lettuce. Hemiustilaginoidin F was active against the five human cancer cells, with half maximal inhibitory concentrations (IC_50_) of 13.2~37.3 μM [11]. Hemiustilaginoidin F, epihemiustilaginoidin D and hemiustilaginoidin D inhibit the growth of pathogenic bacteria with minimum inhibitory concentrations ranging from 8 to 32 µg/mL. Furthermore, hemiustilaginoidin D exhibits antifungal activity against *Magnaporthe oryzae* (IC_50_, 5.21 µg/mL) [11]. Ustilaginoidin D, in particular, has garnered significant attention due to its multiple biological potency. Our previous studies have demonstrated that zebrafish larvae exposed to ustilaginoidin D exhibit delayed yolk sac absorption and impaired development and motor functions [12]. Transcriptome analysis further revealed significant alterations in genes associated with lipid metabolism and liver injury, indicating that ustilaginoidin D exerts strong toxic effects on aquatic model species [12]. However, the precise molecular mechanisms underlying ustilaginoidin D-induced liver injury remain poorly understood. It is unclear whether its toxicity stems from direct mitochondrial dysfunction due to ATP synthesis inhibition or other pathways. This mechanistic ambiguity, coupled with limited data on dose–response relationships and tissue-specific toxicity profiles, highlights the need for further research to elucidate the full scope of the toxicological impact of ustilaginoidin D.

The increasing geographical distribution of rice false smut has raised substantial concerns regarding the environmental accumulation of ustilaginoidins and related mycotoxins within the food chain. While in vitro studies have preliminarily investigated the cytotoxic effects of ustilaginoidin D, critical knowledge gaps remain regarding its comprehensive toxicological profile in mammalian systems. The impacts of ustilaginoidins on behavioral parameters, biological toxicity mechanisms, and gene expression regulation in living organisms are still poorly understood.

Mice have emerged as a crucial mammalian model for investigating biological processes and functions, particularly in toxicity studies [13]. Numerous mycotoxins have been reported to induce liver damage. For example, aflatoxin B1 exhibits substantial hepatotoxicity, causing severe liver damage and even cancer [14]. Similarly, deoxynivalenol (DON) is a prevalent toxin that induces severe liver damage through hepatocellular oxidative stress [15]. Compared to in vitro cell cultures, mice offer distinct advantages in evaluating a broader spectrum of phenotypes. In hepatotoxicity assessment, mouse livers provide functional observability for detailed analysis. Mouse toxicity testing has been established as a standard assay endorsed by the International Standards Organization due to its cost-effectiveness, operational simplicity, and high sensitivity. Notably, these experiments enable the simultaneous recording of multiple toxicity indicators and facilitate exploring toxicity mechanisms [16,17]. The remarkable genomic similarity between humans and mice, with up to 87% sequence homology, further enhances the relevance of the mouse as a model organism. Specifically, both species possess multiple cytochrome P450 enzymes that catalyze the oxidative metabolism of various structurally diverse drugs and toxins [18]. This similarity extends to molecular and cellular processes across multiple organ systems, making mice an ideal model for toxicity identification of ustilaginoidins.

This study demonstrated that ustilaginoidin D is acutely toxic to mice. High doses (225 and 450 mg of ustilaginoidin D/kg·bw) caused significant liver damage and mortality. Additionally, ustilaginoidin D altered the enzyme activities of alanine transaminase (ALT) and aspartate transaminase (AST) and the content of glutathione (GSH) in the liver of mice. Transcriptome analysis further revealed that the differentially expressed genes were significantly enriched in cancer-related pathways, hypertrophic cardiomyopathy and metabolic pathways. These findings highlight the detrimental effects of ustilaginoidin D on animal health and deepen our understanding of its toxicity mechanisms. Importantly, these findings also suggest potential health risks for humans, particularly in the context of food safety and public health.

## 2. Results

### 2.1. Acute Toxicity Induced by Ustilaginoidin D in Mice

**Figure 1:**
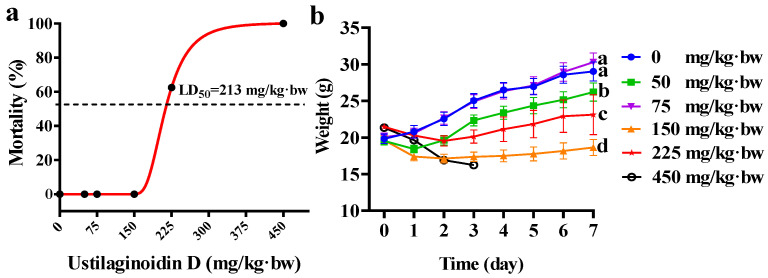
The effects of ustilaginoidin D on mortality and weight in mice. (**a**) The concentration–mortality curve of mice after exposure to different doses of ustilaginoidin D once over 7 d. (**b**) The dynamic change in body weights of the mice treated with varying concentrations of ustilaginoidin D once from 0 to 7 d. Different lowercase letters indicate significant differences in body weight among the treatments with different concentrations of ustilaginoidin D (*p* < 0.05, one-way ANOVA followed by Duncan’s multiple comparison test).

To investigate the toxic effects of ustilaginoidin D on mouse development, the animals were administered various doses of ustilaginoidin D (0, 50, 75, 150, 225, and 450 mg/kg·bw) once via intraperitoneal injection, and continuous monitoring was conducted for seven consecutive days. On the first day after administration, six mice in the highest dose group (450 mg/kg·bw) died, while no mice died in the other groups. On the second day, two mice died in the 225 mg/kg·bw group. On the fourth day, all mice in the highest-dose group (450 mg/kg·bw) died, and three mice in the 225 mg/kg·bw group died (Table S1). No more mice died during the observation period. Finally, 225 mg and 450 mg of ustilaginoidin D/kg·bw resulted in mortality rates of 62.5% and 100%, respectively. Based on these data, an optimal “concentration-mortality” effect curve was modeled, and the LD_50_ value of ustilaginoidin D was determined to be 213 mg of ustilaginoidin D/kg·bw (Figure 1a).

To evaluate the effect of ustilaginoidin D on the body weight of mice, these treated mice were weighed. Within a seven-day monitoring period, the weights of mice treated with 75 mg of ustilaginoidin D/kg·bw showed no significant difference compared to the control group. However, mice treated with 50 mg and 150 mg of ustilaginoidin D/kg·bw exhibited a notable decrease in weight relative to the control group. It is worth noting that mice treated with 225 mg and 450 mg ustilaginoidin D/kg·bw showed weight loss, along with some or all mice dying over seven days (Figure 1b). This suggests that ustilaginoidin D induces acute toxicity in mice and significantly impacts their body weight. Furthermore, acute toxicity tests revealed that mice treated with 150 mg of ustilaginoidin D/kg·bw exhibited behavioral abnormalities, including unstable gait, abdominal pain, and reduced activity (Table S2). These clinical symptoms indicate that the treatment of ustilaginoidin D has seriously impaired the health of the mice, suggesting the acute toxicity of ustilaginoidin D.

### 2.2. Treatment of Ustilaginoidin D Leads to Significant Hepatotoxicity in Mice

**Figure 2:**
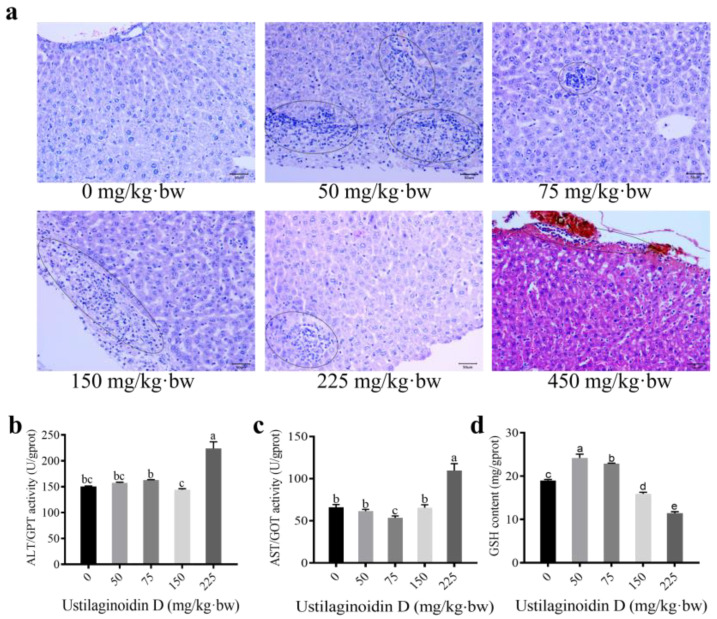
The observation of inflammatory cells and the enzyme activities in mouse liver under ustilaginoidin D treatment. (**a**) HE staining of mouse livers treated with 0, 50, 75, 150, 225, and 450 mg of ustilaginoidin D/kg·bw and 0 mg of ustilaginoidin D/kg·bw, used as the control group. The circles indicate inflammatory cells; bars = 50 μm. (**b**–**d**) Enzyme activities of ALT (**b**), AST (**c**), and the content of GSH (**d**), in ustilaginoidin D-treated mouse livers. Different lowercase letters indicate significant differences among mouse groups with the treatments of varying doses of ustilaginoidin D (*p* < 0.05, one-way ANOVA followed by Duncan’s multiple comparison test).

To evaluate the effects of various doses of ustilaginoidin D on liver lesions in mice, the treated mice were dissected, and the livers were subjected to histopathological examination. Minor inflammatory cell infiltration was observed in the livers of mice in the 50 mg of ustilaginoidin D/kg·bw group. Meanwhile, more pronounced inflammatory cell infiltration was noted in the livers of mice treated with higher doses of ustilaginoidin D (75, 150, 225, and 450 mg of ustilaginoidin D/kg·bw). The results indicate that higher doses of ustilaginoidin D are associated with increased inflammatory cell infiltration, thereby reflecting higher levels of inflammation compared to the control group (Figure 2a). Obviously, these findings showed a clear correlation between ustilaginoidin D concentration and the severity of liver inflammation compared to the control group.

The ALT and AST activities are indicators of liver injury. Glutathione (GSH) is a crucial antioxidant predominantly synthesized in the liver. It plays a vital role in cellular defense mechanisms against oxidative stress and detoxification processes [19]. To assess the degree of liver tissue damage and oxidative stress in mice treated with ustilaginoidin D at various concentrations, the activities of ALT, AST, and the content of GSH were measured. The results showed that the ALT activity was significantly increased in mice after treatment with 225 mg of ustilaginoidin D/kg·bw. The AST activities were significantly reduced after treatments with 75 of ustilaginoidin D/kg·bw, and it was significantly increased following treatment with 225 mg of ustilaginoidin D/kg·bw. On the contrary, the GSH content showed a dual-directional modulation: it was significantly enhanced in the low-dose groups (50 and 75 mg of ustilaginoidin D/kg·bw), whereas it was markedly inhibited in the high-dose groups (150, and 225 of ustilaginoidin D/kg·bw) (Figure 2b–d). These findings suggest that the mouse liver injury caused by ustilaginoidin D exhibits a nonlinear dose–response relationship. Specifically, medium and low doses (≤150 mg of ustilaginoidin D/kg·bw) primarily induce inflammatory responses and activate compensatory antioxidant mechanisms. In contrast, high doses (≥225 mg of ustilaginoidin D/kg·bw) lead to the breakdown of the antioxidant system, characterized by significant increases in infiltrating inflammatory cells and irreversible liver injury.

### 2.3. Comprehensive Transcriptome Analysis to Evaluate the Impact of Ustilaginoidin D on the Mouse Liver Function

**Figure 3:**
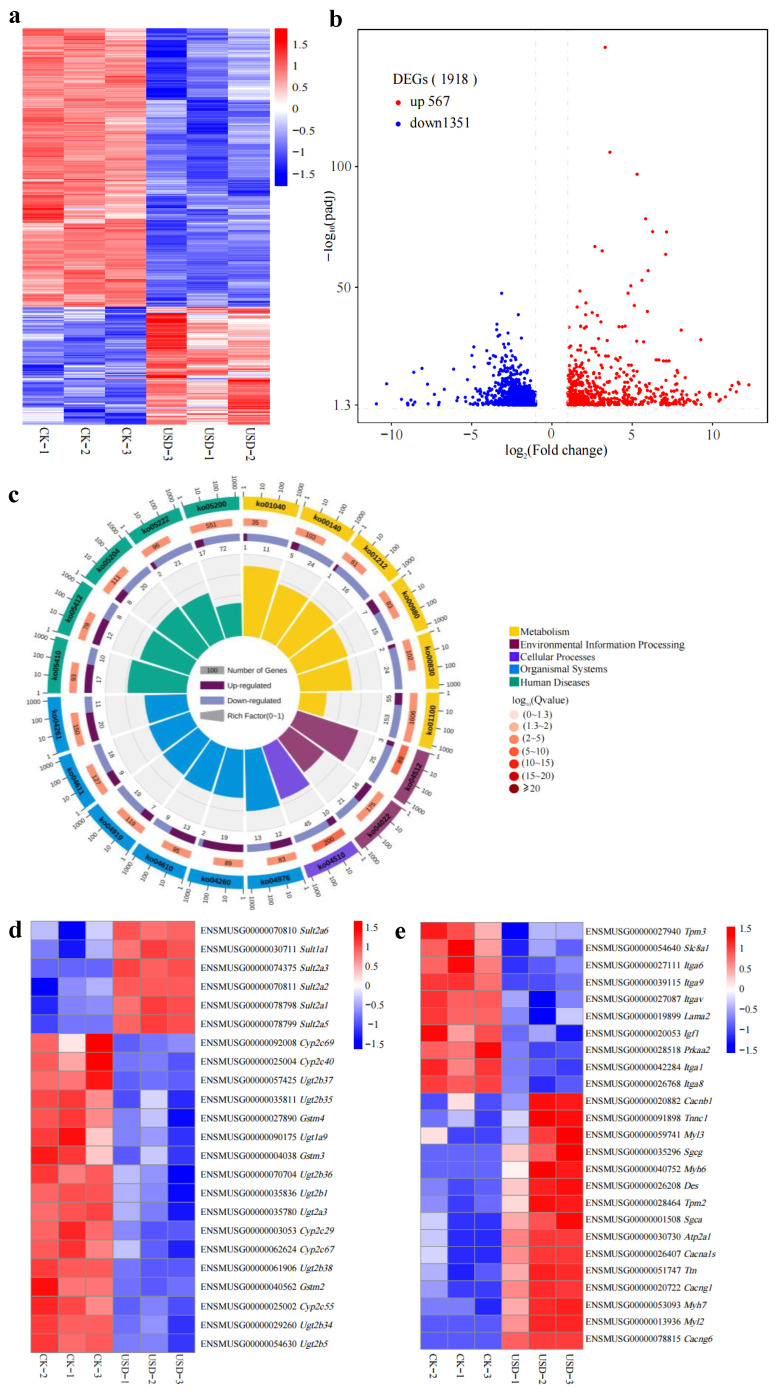
Transcriptome analysis of mouse liver after exposure to ustilaginoidin D. (**a**) Heatmap analysis to show the expression levels of DEGs in mouse liver upon ustilaginoidin D (USD) treatment. Red and blue represent up-regulated and down-regulated genes, respectively. (**b**) The volcano plot was graphed according to DEGs in mouse liver upon ustilaginoidin D (USD) treatment. Red and blue represent up-regulated and down-regulated genes, respectively. (**c**) KEGG pathway enrichment circle diagram: the first circle consists of the top 20 enrichment pathways, and the coordinates of the number of differential genes outside the circle are shown. Different colors represent different pathways. The number of differentially expressed genes and the Q value for this pathway influence its representation. A higher count of DEGs results in a longer bar, while a lower Q value produces a redder color. The second circle indicates the number of genes in this pathway. The third circle indicates the number of DEGs, where dark purple represents the number of up-regulated DEGs, and light purple represents the number of down-regulated DEGs. The fourth circle indicates the Rich Factor value of each pathway (the number of DEGs in this pathway divided by all the numbers in this pathway) and background grid lines, with each grid representing 0.1. (**d**,**e**) Heatmap analysis to show the expression levels of chemical carcinogenesis (**d**) and hypertrophic cardiomyopathy (HCM) (**e**) pathway genes.

To investigate the toxicological mechanisms of ustilaginoidin D on the mice hepatic systems, transcriptomic profiling was performed by analyzing liver tissues collected from the control group and the group treated with 150 mg of ustilaginoidin D/kg·bw. High-quality RNA-Seq data (Q30 > 91%, Q20 > 96%) and distinct PCA clustering patterns (Figure S1) confirmed robust group segregation, demonstrating treatment-specific transcriptional reprogramming. Comparative transcriptomic profiling of hepatic tissues (*n* = 3 per group) identified 1918 differentially expressed genes (DEGs), including 567 up-regulated and 1351 down-regulated genes (FDR < 0.05, |log_2_FC| > 1), upon ustilaginoidin D exposure (Figure 3a,b). KEGG pathway analysis revealed that the DEGs were significantly enriched in Metabolism, Environmental Information Processing, Cellular Processes, Organismal Systems and Human Diseases.

Metabolism encompasses a variety of crucial biological processes, including the biosynthesis of unsaturated fatty acids (ko01040), steroid hormone biosynthesis (ko00140), fatty acid metabolism (ko01212), metabolism of xenobiotics by cytochrome P450 (ko00980), retinol metabolism (ko00830), and the comprehensive metabolic pathways (ko01100). These pathways play essential roles in maintaining the physiological functions and metabolic equilibrium of living organisms. Human Diseases involves several key pathways, such as hypertrophic cardiomyopathy (HCM, ko05410), arrhythmogenic right ventricular cardiomyopathy (ARVC, ko05412), chemical carcinogenesis (ko05204), small cell lung cancer (ko05222), and general cancer pathways (ko05200). Environmental Information Processing includes the cGMP-PKG signaling pathway (ko04022) and ECM–receptor interactions (ko04512). Cellular Processes highlights focal adhesion mechanisms (ko04510). Organismal Systems encompasses multiple functions: adrenergic signaling in cardiomyocytes (ko04261), platelet activation (ko04611), thyroid hormone signaling (ko04919), complement and coagulation cascades (ko04610), cardiac muscle contraction (ko04260), and bile secretion (ko04976) (Figure 3c). Heatmap analysis of ko05204 (chemical carcinogenesis) pathways revealed the coordinated up-regulation of sulfotransferase genes along with down-regulation of cytochrome P450 enzymes, UDP-glucuronosyltransferases, and glutathione S-transferase regulator genes (Figure 3d,e). The results indicate that the mice treated with ustilaginoidin D treatment exhibit significant changes in the gene expression profile of livers, especially with notable enrichment in multiple Metabolism- and Human-Diseases-related pathways.

### 2.4. Validation of Differentially Expressed Genes by RT-qPCR

**Figure 4:**
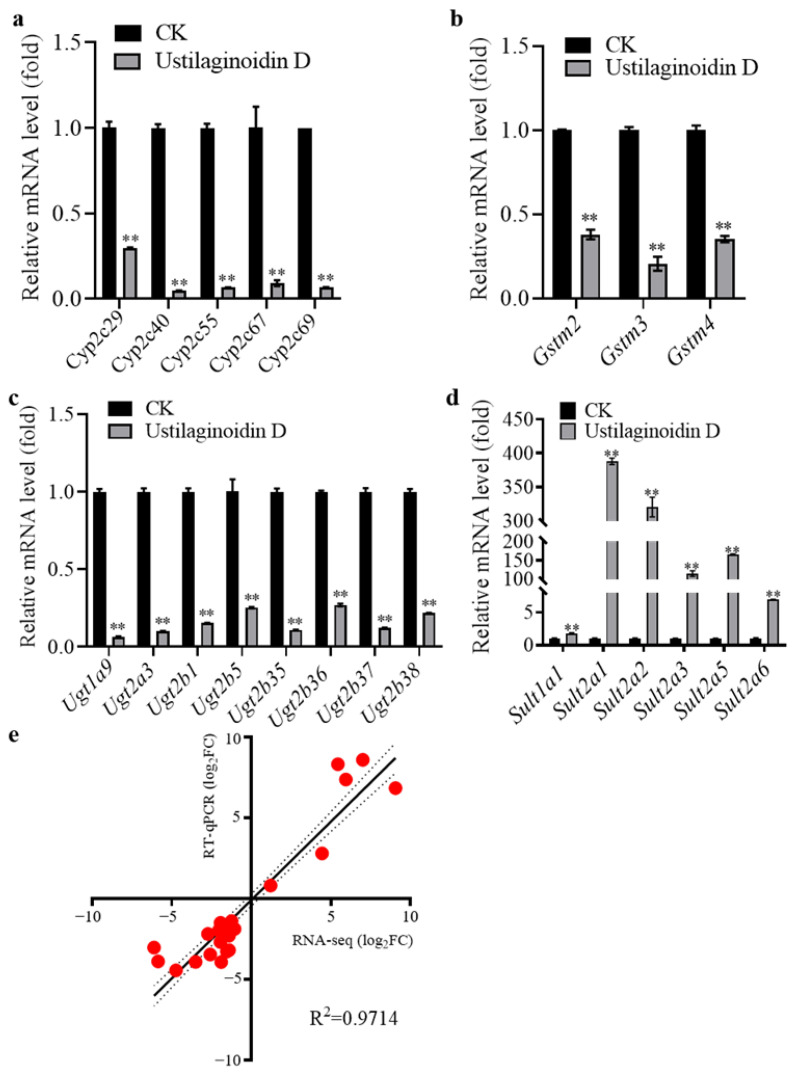
RT-qPCR assay to validate differentially expressed genes in mice after ustilaginoidin D exposure. (**a**–**d**) RT-qPCR assays to detect the expression levels of twenty-two genes that are related to different pathways in mouse liver with or without ustilaginoidin D exposure. (**a**) Cytochrome P450 enzyme genes, (**b**) glutathione S-transferase genes, (**c**) UDP-glucuronosyltransferase genes, and (**d**) sulfotransferase genes. The *GAPDH* gene was used as an internal reference. The data are presented as mean ± standard deviation. The significance of the difference was determined by paired *t*-tests. ** (*p* < 0.05) indicate significant differences in the gene expression levels between CK and ustilaginoidin D exposure. (**e**) Correlation analysis between RT-qPCR data and RNA-seq-derived gene expression profiles.

To validate the accuracy of transcriptome analysis, we selected 22 differentially expressed genes (DEGs) related to cytochrome P450 enzymes, UDP-glucuronosyltransferases, sulfotransferase, and glutathione S-transferases for RT-qPCR validation. The results demonstrated that 16 out of the 22 genes associated with cytochrome P450 enzymes (*Cyp2c55*, *Cyp2c29*, *Cyp2c67*, *Cyp2c40*, *Cyp2c69*), UDP-glucuronosyltransferases (*Ugt1a9*, *Ugt2b1*, *Ugt2b35*, *Ugt2b36*, *Ugt2b5*, *Ugt2b37*, *Ugt2a3*, *Ugt2b38*), and glutathione S-transferases (*Gstm3*, *Gstm2*, *Gstm4*) were significantly down-regulated following treatment with ustilaginoidin D (Figure 4a–c). In contrast, the remaining six genes, which are associated with sulfotransferases (*Sult2a2*, *Sult2a1*, *Sult2a3*, *Sult2a6*, *Sult1a1*, *Sult2a5*) exhibited significant up-regulation patterns (Figure 4d). Meanwhile, a comparative analysis was performed to assess the correlation between RT-qPCR and RNA-seq data (Figure 4e). The scatter plot illustrates a robust positive correlation between the RT-qPCR data and RNA-seq-derived gene expression profiles, as evidenced by an impressive R² value of 0.9714 (Figure 4e), indicating that transcriptome analysis was reliable.

## 3. Discussion

A previous study demonstrated that the intraperitoneal administration of *U. virens*-derived crude extracts (U-3) and purified ustiloxin crystals induces acute necrosis in isolated hepatocytes and renal tubular cells, confirming direct hepatorenal toxicity followed by systemic mitotic damage [20]. Emerging evidence reveals that ustilaginoidin D exhibits potent hepatotoxicity in aquatic models and impairs locomotor behavior in zebrafish [12]. The food safety implications of ustilaginoidin D contamination in rice demand urgent attention, given its potential threat to human and animal health. However, critical gaps remain regarding exposure thresholds, dose–response relationships, and preventive strategies for ustilaginoidin D toxicity. 

In this study, we investigated the toxic effects of different doses of ustilaginoidin D on mice. The results demonstrated that ustilaginoidin D exhibited dose-dependent hepatotoxicity, characterized by inflammatory cell infiltration at the hepatic surface (Figure 2a). Notably, mortality was observed in mice treated with high doses of ustilaginoidin D (Figure 1a). Exposure to ustilaginoidin D significantly alters the activity of ALT and AST, and also the content of GSH (Figure 2b–d), a critical antioxidant essential for maintaining normal immune function in mouse liver [21]. This suggests that ustilaginoidin D may disrupt cellular redox homeostasis, leading to liver damage. These findings indicate that ustilaginoidin D exerts potent hepatotoxic effects and disrupts immune functionality in mice. Furthermore, acute toxicity tests revealed that mice treated with 150 mg of ustilaginoidin D/kg·bw exhibited behavioral abnormalities, including unstable gait, abdominal pain, and reduced activity (Table S2). These typical symptoms [22] before death indicate that ustilaginoidin D caused acute toxicity on mice. This study found that in the livers of mice, there was a significant infiltration of inflammatory cells at a dose of 225 mg of ustilaginoidin D/kg·bw (Figure 2a), which might be related to abdominal pain and reduced activity [23].

Aflatoxin B1 (AFB1) is a well-known mycotoxin produced by *Aspergillus fungi*, which can cause liver damage and has strong carcinogenic properties. AFB induces hepatotoxicity through mitochondrial dysfunction, oxidative stress and inflammation as the central pathological mechanisms [24,25,26]. As mentioned earlier, ustilaginoidin A can inhibit the synthesis of ATP in mitochondria in mouse embryos. We speculate that ustilaginoidin D, as a derivative to ustilaginoidin A, might have a similar mechanism to cause hepatotoxicity. To further clarify molecular mechanisms underlying the hepatotoxicity, transcriptomic analysis reveals significant changes in the expression of detoxification enzyme gene families. The majority of DEGs (15/22) in the Metabolism of xenobiotics by cytochrome P450 (ko00980) of Metabolism exhibited a down-regulation pattern. These enzymes are directly related to the biotransformation pathways of drugs and toxins and play a crucial role in the detoxification process of liver. Specifically, cytochrome P450 enzymes are responsible for the oxidative metabolism of xenobiotics, and their reduction leads to toxin accumulation [27]. In addition, Ugt-mediated glucuronidation is a key step in the excretion of drugs and toxins, and glutathione S-transferases catalyze the detoxification of electrophilic substances by conjugation with GSH [28]. Currently, detoxification enzyme family genes, such as the *GST* family (such as *GSTA4*), have been reported to affect the detoxification efficiency of AFB1 [29]. Moreover, two key metabolic enzymes in humans, glutathione S-transferase (GST) M1 and GSTT1, play a crucial role in detoxifying AFB1 by catalyzing the coupling of GSH with aflatoxin B1-epoxide (AFBO) [30,31,32,33,34,35,36]. In the transcriptome, the down-regulation of these genes is directly linked to a significant decrease in GSH content in the high-dose group (≥150 mg of ustilaginoidin D/kg·bw), indicating a failure of the antioxidant system. These findings provide a molecular basis for ustilaginoidin D-induced hepatotoxicity. However, ustilaginoidin D induces the expression of six sulfotransferase genes (Figure 4d). Sulfotransferases play crucial roles in detoxification through catalyzing the sulfation of xenobiotics, including pharmaceuticals and environmental pollutants and oxidative stress regulation [37]. These results demonstrate that ustilaginoidin D disrupts xenobiotic oxidative metabolism by simultaneously down-regulating detoxification enzyme genes (cytochrome P450 genes, UDP-glucuronosyltransferase genes, and glutathione S-transferase genes) while up-regulating the expression of sulfotransferase genes, ultimately leading to hepatocyte damage. It highlights the necessity for focused interventions to reduce their detrimental impacts on liver injury caused by mycotoxins [38,39,40]. As revealed by the transcriptome data, differentially expressed genes in mouse liver tissues after ustilaginoidin D treatment are significantly enriched in the pathways associated with cancer, hypertrophic cardiomyopathy, small cell lung carcinoma (SCLC), and cardiovascular diseases. *MYH7* are well-known causes of hypertrophic cardiomyopathy (HCM) [41,42]. *ITGAV* serves as a potential marker for the prognosis and identification of cancers, including SCLC [43]. The role of *ITGAV* in promoting cancer progression has been experimentally confirmed in hepatocellular carcinoma and pancreatic cancer [44]. In this study, the expression of *MYH7* was up-regulated in the liver tissues of mice treated with ustilaginoidin D, indicating that mice treated with ustilaginoidin D have a risk of heart disease, and the down-regulation of *ITGAV* may be closely related to cancer markers. Furthermore, the detoxification enzyme family genes have been reported to not only play the role of metabolizing exogenous drugs, but also be closely related to the occurrence of cancer [45]. Furthermore, the two human homologous genes of *Cyp2c29*, *Cyp2C8* and *Cyp2C9*, are down-regulated during the progression of human hepatocellular carcinoma [46]. Compared with normal bladder tissues (ANBTs), the expression of *SULT1A2* in bladder cancer tissues is higher [47]. In addition, after treating the livers of mice with 150 mg ustilaginoidin D/kg·bw, the detoxification enzyme genes were differentially expressed in the transcriptome (Figure 4b), indicating that treating mice with ustilaginoidin D may lead to metabolic defects of detoxification enzymes in the livers of mice. This leads to a decrease in the metabolic capacity of exogenous detoxification enzymes, thereby increasing the risk of cancer [45]. Futhermore, our transcriptome data showed tha 98 out of 243 DEGs in cancer-related pathways of human diseases exhibited down-regulation patterns (Table S3). This indicates that ustilaginoidin D might be closely related to cancinogenesis or have an anti-cancer effect. Low doses of ustilaginoidin F have been previously demonstrated to have an anti-cancer activity with significant inhibitory effects in five human cancer cell lines [11]. Therefore, it is of great interest to investigate how ustilaginoidin D affects human cancer at different doses in the future.

In summary, after mice were administered with ustilaginoidin D via intraperitoneal injection, distinct toxic reactions, such as weight loss, liver damage, and mortality, were observed across different dose groups. However, this administration route differs significantly from the primary exposure route of ustilaginoidins to humans. Humans are mainly exposed to ustilaginoidin D through contaminated food, especially grain products, where exposure occurs orally, not via intraperitoneal injection. For orally ingested compounds, changes in biotransformation enzyme expression can influence their metabolism. Future studies should therefore focus on the impact of systemic pre-clearance and the liver’s first-pass effect on ustilaginoidin D toxicity. These findings demonstrate that ustilaginoidin D is a potent mycotoxin with strong hepatotoxicity and potential carcinogenic-promoting effects that poses significant risks to food safety and public health. The study highlights the need for further research to elucidate the mechanisms of ustilaginoidin D-induced toxicity and to develop effective strategies for mitigating its impact on human and animal health.

## 4. Conclusions

This study demonstrates that ustilaginoidin D induces significant hepatotoxicity in mice, characterized by histopathological alterations in liver architecture and the dysregulation of metabolism pathways. Furthermore, ustilaginoidin D triggers the up-regulation of genes associated with oxidative stress, inflammatory responses, and hepatocellular injury. These findings underscore the potent hepatotoxic effects of ustilaginoidin D in mammalian systems, emphasizing its potential risks to food safety and public health. The results may contribute to the establishment of safety thresholds for ustilaginoidin D in food products and provide a robust mammalian model for investigating the toxicological mechanisms of bis-naphtho-γ-pyrone mycotoxins. This study also highlights the need for further research into the long-term effects and molecular pathways underlying ustilaginoidin D-induced hepatotoxicity in mammals.

## 5. Materials and Methods

### 5.1. Experimental Animals, Diets, and Housing Conditions

Fifty-six specific pathogen-free six-week-old KM (KunMing) mice were obtained from Jinan Pengyue Laboratory Animal Breeding Co., Ltd., Jinan, China, comprising equal numbers of males and females, with body weights ranging from 18 to 22 g. The mice were provided with a standardized diet, endorsed by the quality certification number No. 120210802011 from Jiangsu Province Synergy Pharmaceutical Biological Engineering Co., Ltd., Nanjing, China, which holds the production license number Su Feed Certificate (2019) 01008. The bedding consisted of experimental corncob packing material identified by the quality certification number No. 120210623018 from Jiangsu Sye Pharmaceutical Biological Engineering Co., Ltd., Nanjing, China, The animals were housed within a controlled barrier facility, certified under license number SYXK (Lu) 20210015, where both temperature and relative humidity were meticulously maintained at 20–26 °C and 40–70%, respectively. Standardized conditions were established to ensure optimal animal welfare, minimize environmental variability, and support the reproducibility of experimental outcomes.

### 5.2. Chemicals, Reagents, Treatment and Dosing Protocols

Ustilaginoidin D was extracted and purified from *U. virens* as previously described. HPLC (LC-20A Shimadzu, Kyoto, Japan)was performed to purify ustilaginoidin D and to determine its purity (Figure S2a,b) [48,49,50]. Ustilaginoidin D (≥98% purity) was dissolved in absolute ethanol (≥99.8% purity) to prepare a stock solution. The stock solution was then diluted with normal saline to achieve the desired concentration. The dosages of the treatment groups were 50, 75, 150, 225, and 450 mg of ustilaginoidin D/kg·bw, respectively. Mice were weighed before exposure to ustilaginoidin D and were then intraperitoneally injected at a volume of 0.1 mL/10 g·bw to achieve the desired dose. Sterile normal saline was used as a control. The mice were administered via intraperitoneal injection only once, and continuous monitoring was conducted for seven consecutive days. The body weight of the mice was measured daily. After seven days, all surviving mice were sacrificed by the cervical dislocation method, and their livers were excised. Mice that died prior to the seven-day mark were dissected immediately upon death to retrieve their livers, with the time of liver collection recorded. The liver tissues were preserved at −80 °C for subsequent analysis.

### 5.3. Histopathological Evaluation of Mouse Liver Through Hematoxylin-Eosin Staining

At least three livers were randomly selected from the eight mice in each treatment group, followed by rinsing with sterile 0.9% physiological saline to remove residual blood. The liver lobes were carefully excised for hematoxylin–eosin staining. Tissue specimens were subsequently fixed in 10% neutral buffered formalin (pH 7.4) for 24–48 h at 4 °C to ensure optimal preservation of cellular architecture. The tissues were then processed for histological examination using standard hematoxylin and eosin (H&E) staining protocols [51,52,53,54,55]. The procedure consisted of the following sequential steps: (1) paraffin removal through xylene immersion, (2) rehydration via gradient concentrations of ethanol, (3) nuclear staining with Mayer’s hematoxylin, (4) differentiation in acid alcohol, (5) bluing in Scott’s solution, (6) cytoplasmic counterstaining with eosin Y, (7) dehydration through gradient concentrations of ethanol, (8) clearing in xylene, and (9) mounting with neutral balsam [55,56]. For each specimen, three sections were selected for staining. Histopathological evaluation was performed using a light microscope equipped with a digital imaging system (Leica, Wetzlar, Germany, magnification: 100×, 200×, and 400×), and high-resolution micrographs were captured for subsequent quantitative and qualitative analyses.

### 5.4. Assessment of the Enzymatic Activities of ALT and AST and the Content of GSH in Mouse Livers

The activities of ALT and AST and the content of GSH in the liver were determined as previously described [57,58,59,60]. Briefly, the liver tissues of mice were removed at −80 °C and quickly frozen in liquid nitrogen and ground into powder. The homogenate was created by adding 9 volumes of normal saline to the tissue powder and was then centrifuged at 4 °C and 2500 rpm for 10 min. The supernatant was collected to determine the activities of ALT and AST and the content of GSH with three technical repeats using the alanine aminotransferase (ALT/GPT), aspartate aminotransferase (AST/GOT), and glutathione (GSH) assay kits according to the manufacturer’s instructions (Nanjing Jiancheng Bioengineering Institute, Nanjing, China).

### 5.5. RNA-Seq Analysis

The livers from mice groups treated with 0 and 150 mg/kg·bw ustilaginoidin D were used for transcriptomic analysis. Specifically, the entire harvested livers were rapidly frozen in liquid nitrogen and used for transcriptome analysis. Total RNAs were extracted from the collected livers using a Trizol reagent kit (Invitrogen, Carlsbad, CA, USA), and RNA quality was assessed on an Agilent Bioanalyzer 2100 (Agilent Technologies, Palo Alto, CA, USA). Total RNAs were reversely transcribed into cDNA using an NEBNext Ultra RNA Library Prep Kit for Illumina (New England Biolabs, Ipswich, MA, USA). The cDNA library construction and sequencing were performed at Gene Denovo Biotechnology Co., Ltd. (Guangzhou, China) using an Illumina Novaseq6000.

### 5.6. Quantitative Real-Time PCR

Twenty-two differentially expressed genes associated with Cytochrome P450s, UDP-glucuronosyltransferases and Glutathione S-transferases were selected and detected by quantitative real-time PCR (RT-qPCR). Total hepatic RNAs were extracted using an Ultrapure RNA Kit (CWBIO, Beijing, China). Total RNAs (2 μg) were reversely transcribed into cDNA using a PrimeScript^TM^ reverse transcription reagent kit (TAKARA, Otsu, Shiga, Japan) following the manufacturer’s instruction. The qPCR primers were designed via Primer-BLAST (https://www.ncbi.nlm.nih.gov/tools/primer-blast/, accessed on 5 July 2022) and are listed in Table S4. RT-qPCR was performed using Fast SYBR mixture (CWBIO, Jiangsu, China) with a LightCycler^®^ 96 system (Roche, Risch-Rotkreuz, Switzerland) as described previously [12]. The expression of *Mus musculus GAPDH* (encoding GAPDH) was used as an internal control. Relative expression was calculated using the 2^−∆∆CT^ method [61,62].

### 5.7. Statistical Analyses

Data processing was conducted using SPSS Statistics 26 (IBM Corp., Armonk, NY, USA). All data are presented as means ± standard deviation (SD). One-way ANOVA followed by Duncan’s multiple comparison test was employed to evaluate the significance of differences among groups, with a *p*-value of less than 0.05 considered statistically significant.

## Data Availability

The original contributions presented in this study are included in this article. Further inquiries can be directed to the corresponding authors.

## Supplementary Materials

The following supporting information can be downloaded at https://www.mdpi.com/article/10.3390/toxins17050250/s1. Figure S1: Sample principal component analysis; Figure S2: The purification and chemical structure of ustilaginoidin D; Table S1: Survival status of mice during the administration process; Table S2: Liver histopathology findings; Table S3: The number of up-regulated and down-regulated cancer-associated DEGs; Table S4: Primer for RT-qPCR.
