# Supplementary material for: Ustilaginoidin D Induces Acute Toxicity and Hepatotoxicity in Mice
Source: Toxins (Basel). 2025 May 17;17(5):250. doi: 10.3390/toxins17050250 (PMC12115440; doi:10.3390/toxins17050250)
Supplement: Supplementary file 1 [file toxins-17-00250-s001.zip › toxins-3613504-supplementary.pdf]

Supplementary Materials: Ustilaginoidin D Induces Acute Toxicity and Hepatotoxicity in Mice

Bo Wang, Xiaolong Bai, Min Zhang, Xiangxiang Liu, Muhammad Zulqar Nain Dara, Lingjing Liu, Ming-ming Ou, Dayong Li, Jiyang Wang, Ling Liu and Wenxian Sun

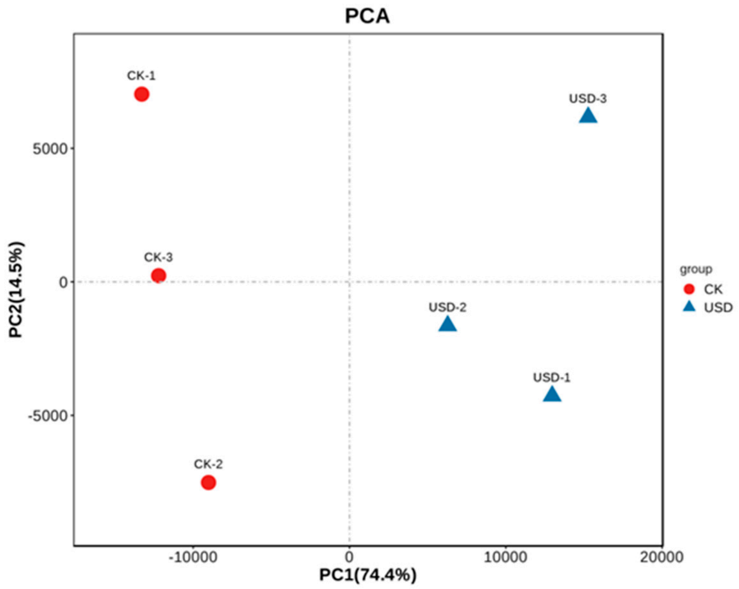

Figure S1. Sample principal component analysis. The PC1 coordinate represents the first principal component, and the percentage in brackets represents the contribution value of the first principal component to the sample difference. The PC2 coordinate represents the second principal component, and the percentage in brackets represents the contribution value of the second principal component to the sample difference. The colored points in the figure represent each.

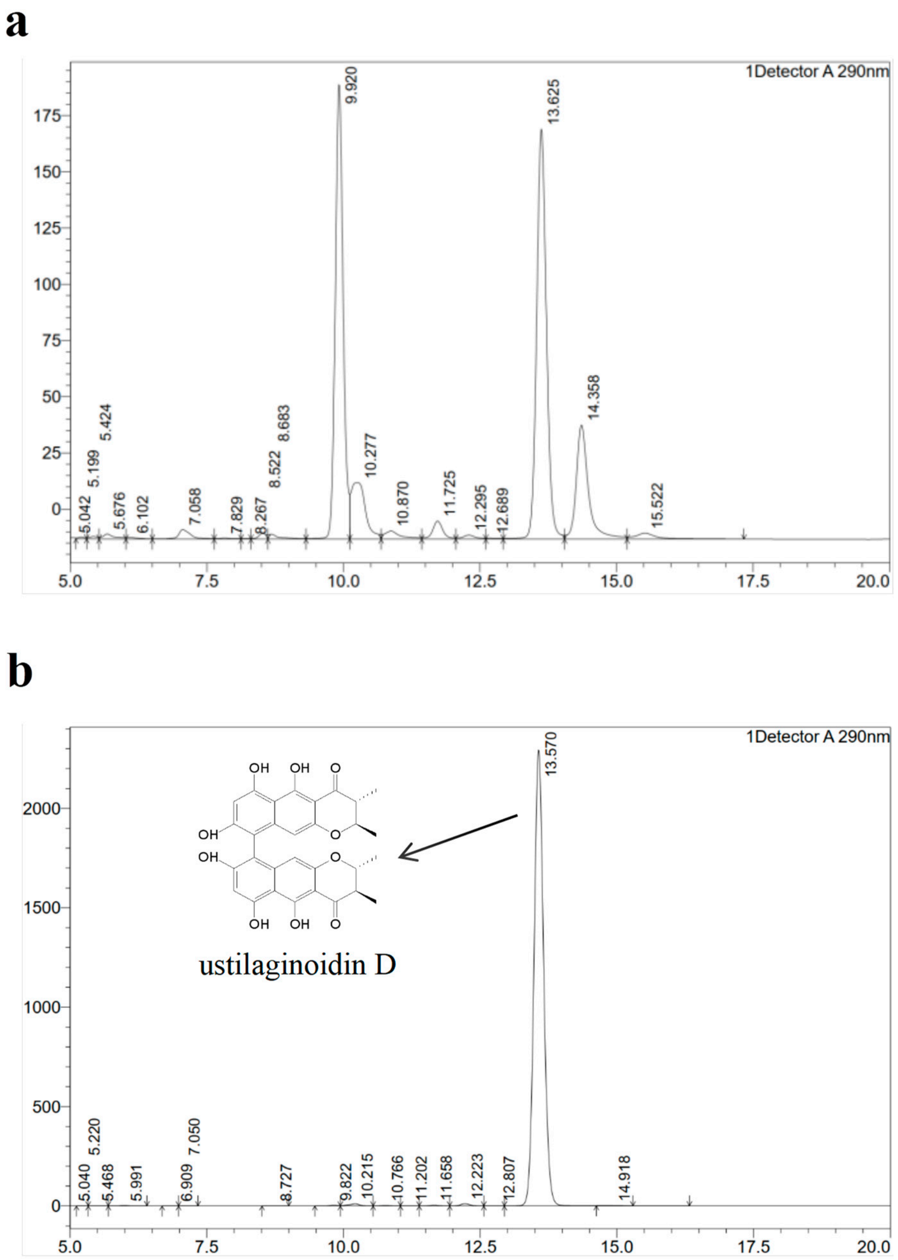

Figure S2. The purification and chemical structure [11] of ustilaginoidin D. HPLC profiles of ustilaginoidin derivatives before purification of ustilaginoidin D. The purity of ustilaginoidin D after the purification and its chemical structure.

Table S1. Survival status of mice during the administration process.

Dose (mg of usti-laginoidin D/kg·bw)	Gender	Number	D0	D1	D2	D3	D4	D5	D6	D7
225	♀	7141	Alive	Alive	Alive	Alive	Alive	Alive	Alive	Alive
		7142	Alive	Alive	/	/	/	/	/	/
		7143	Alive	Alive	Alive	Alive	/	/	/	/
		7144	Alive	Alive	Alive	Alive	Alive	Alive	Alive	Alive
	♂	7145	Alive	Alive	Alive	Alive	/	/	/	/
		7146	Alive	Alive	/	/	/	/	/	/
		7147	Alive	Alive	Alive	/	/	/	/	/
		7148	Alive	Alive	Alive	Alive	Alive	Alive	Alive	Alive
450	♀	6691	Alive	Alive	/	/	/	/	/	/
		6692	Alive	/	/	/	/	/	/	/
		6693	Alive	/	/	/	/	/	/	/
		6694	Alive	/	/	/	/	/	/	/
	♂	6695	Alive	/	/	/	/	/	/	/
		6696	Alive	/	/	/	/	/	/	/
		6697	Alive	/	/	/	/	/	/	/
		6698	Alive	Alive	Alive	Alive	/	/	/	/

Note: The "/" mean dead.

Table S2. Liver Histopathology Findings.

Dose (mg of usti-laginoidin D/kg·bw)	Animal number	Histological findings	Degree of le-sion
0	7101	No obvious abnormality	-
	7102	No obvious abnormality	-
	7103	No obvious abnormality	-
	7104	No obvious abnormality	-
	7105	No obvious abnormality	-
	7106	No obvious abnormality	-
	7107	No obvious abnormality	-
	7108	No obvious abnormality	-
50	7117	No obvious abnormality	-
	7118	Focal hepatocyte necrosis, inflammatory cell infiltration and multiple inflammatory granulomas were observed at the liver margin	±
	7119	No obvious abnormality	-
	7120	Multifocal hepatocyte necrosis, accompanied by inflammatory cell infiltration, fibrin deposition on the liver surface, and inflam-matory cell infiltration	+
	7121	Multifocal inflammatory cell infiltration and formation of multi-ple inflammatory granulomas	±
	7122	Multifocal inflammatory cell infiltration and formation of multi-ple inflammatory granulomas	±
	7123	Multifocal inflammatory cell infiltration and formation of multi-ple inflammatory granulomas	±

75	7124	Focal hepatocyte necrosis, accompanied by inflammatory cell infiltration, fibrin deposition on the liver surface, and inflammatory cell infiltration	+
	7125	Multifocal inflammatory cell infiltration	±
	7126	Multifocal hepatocyte punctate necrosis, accompanied by inflammatory cell infiltration	±
	7127	No obvious abnormality	-
	7128	Formation of multiple inflammatory granulomas	±
	7129	Multifocal inflammatory cell infiltration and formation of multiple inflammatory granulomas	±
	7130	No obvious abnormality	-
	7131	Hepatocyte punctate necrosis	±
	7132	No obvious abnormality	-
150	7133	Multifocal inflammatory cell infiltration, multiple inflammatory granulomas formation, extensive fibrin deposition on the liver surface and accompanying inflammatory cell infiltration	+
	7134	Multifocal inflammatory cell infiltration, multiple inflammatory granulomas formation, extensive fibrin deposition on the liver surface and accompanying inflammatory cell infiltration	+
	7135	Multifocal hepatocyte necrosis, multifocal inflammatory cell infiltration, inflammatory granuloma formation, extensive fibrin deposition on the liver surface and accompanying inflammatory cell infiltration	+
	7136	Multifocal hepatocyte necrosis, accompanied by inflammatory cell infiltration and fibrin deposition on the liver surface	++
	7137	Multifocal hepatocyte necrosis, with inflammatory cell infiltration, multifocal inflammatory cell infiltration within the liver, and fibrin deposition on the liver surface	±
	7138	Multifocal hepatocyte necrosis, with inflammatory cell infiltration, multifocal inflammatory cell infiltration within the liver, and fibrin deposition on the liver surface	+
	7139	Multifocal hepatocyte necrosis, with inflammatory cell infiltration, multifocal inflammatory cell infiltration within the liver, and fibrin deposition on the liver surface	+
	7140	Multifocal hepatocyte necrosis, accompanied by inflammatory cell infiltration and multiple inflammatory granuloma formation	+
225	7141	Multifocal hepatocyte necrosis, accompanied by inflammatory cell infiltration and fibrin deposition on the liver surface	++
	7142	No obvious abnormality	±
	7143	No obvious abnormality	-
	7144	Focal hepatocyte necrosis, with inflammatory cell infiltration and fibrin deposition on the liver surface	+
	7145	Diffuse hepatocyte necrosis, with inflammatory cell infiltration and fibrin on the liver surface	++
	7146	Multifocal hepatocyte necrosis, with extensive fibrin deposition on the liver surface	++
	7147	Fibrin deposition on the liver surface, accompanied by inflammatory cell infiltration	+
	7148	Multifocal hepatocyte necrosis, with inflammatory cell infiltration and extensive fibrin on the liver surface	+

450	6691	Hepatocyte necrosis at the liver margin, with cellular debris and inflammatory cell infiltration within the necrotic foci	+
	6692	Fibrin deposition on the liver surface, accompanied by inflammatory cell infiltration	±
	6693	Surface fibrin deposition with inflammatory cell infiltration	±
	6694	Multifocal hepatocyte necrosis, with inflammatory cell infiltration, scattered individual hepatocyte necrosis, and fibrin on the liver surface	±
	6695	Fibrin deposition on the liver surface, accompanied by inflammatory cell infiltration	±
	6696	Fibrin deposition on the liver surface, accompanied by inflammatory cell infiltration	±
	6697	No obvious abnormality	-
	6698	Multifocal necrosis in the hepatic cells at the liver margin, with inflammatory cells and necrotic cell debris visible within the necrotic foci A large amount of fibrin is attached to the surface of the liver	+

Note: The lesion grades are classified as follows: No obvious abnormality（-）, Minimal (±), Mild (+), Moderate (++), Severe (+++).

Table S3. The number of upregulated and downregulated cancer-associated DEGs.

KEGG_A_class	KEGG_B_class	Pathway	CK-vs-USD (905)	All (9221)	Pvalue	Pathway ID	Up	Down
Human Diseases	Cancers	Pathways in cancer	89	551	1.06E-06	ko05200	17	72
Human Diseases	Cancers	Chemical carcinogenesis	28	111	1.98E-06	ko05204	8	20
Human Diseases	Cancers	Proteoglycans in cancer	36	211	0.000661583	ko05205	11	25
Human Diseases	Cancers	MicroRNAs in cancer	32	167	0.000155569	ko05206	2	30
Human Diseases	Cancers	Basal cell carcinoma	12	63	0.01808137	ko05217	2	10
Human Diseases	Cancers	Small cell lung cancer	23	96	3.92E-05	ko05222	2	21
Human Diseases	Cancers	Breast cancer	23	149	0.01883352	ko05224	3	20

Table S4. Primer for RT-qPCR.

Primer name	Primer sequence (5'-3')
Cyp2c55-Forward primer	CCCCAAGGGCACAGAGTTAGT
Cyp2c55-Reverse primer	CTCTCCCACGCACATTCGCT
Cyp2c29-Forward primer	ACGGATTTGTGCTGGAGAGG
Cyp2c29-Reverse primer	GTGGCAGAGAGGCAAATCCA
Cyp2c67-Forward primer	AAGCACACACACATCACAGC
Cyp2c67-Reverse primer	ATCGTTGGACCTCATGCACC
Cyp2c40-Forward primer	CTCCTGGCCCTACTCCTCTC
Cyp2c40-Reverse primer	TCTCCATGGTCAATGAGGGC
Cyp2c69-Forward primer	GGTGCATGAGGTCCAACGAT
Cyp2c69-Reverse primer	CAGGGTCAAACACCTCTGGG
Ugt1a9-Forward primer	TGTAGCCTTTCCCACCTCCTTTT
Ugt1a9-Reverse primer	CTGCCAACTCACCTCTGGGA
Ugt2b1-Forward primer	TCCCTGTGGTGAGCTGCTAT

Ugt2b1-Reverse primer	AGGCCCCCACTGTACTTTTC
Ugt2b35-Forward primer	CTTGCACACAACCTCACCTG
Ugt2b35-Reverse primer	CAGCACTTTACAGGAAGGACTGC
Ugt2b5-Forward primer	TTATGCGCCACAAAAGAGCC
Ugt2b5-Reverse primer	AGTGGTTGCCACACAAGAGAG
Ugt2b36 Forward primer	GATGGACTTATGAGGTTCCAAGAGA
Ugt2b36 Reverse primer	CTCCCCACAGGGACCAATAG
Ugt2b37-Forward primer	GTACAGTCTTCGCTTCACTGC
Ugt2b37-Reverse primer	AGGCATCTGGAACCAAAAGTCA
Ugt2a3-Forward primer	GAGCCAAGCATCTTCGTGTG
Ugt2a3-Reverse primer	CGACACACAACAATAGGAACC
Ugt2b38-Forward primer	ATGCGCCACAAAAGGGCTAA
Ugt2b38-Reverse primer	ACACAAGAGAGTAGGAAGCCG
Gstm3-Forward primer	TGCTGCAGTCCCGATTTTGA
Gstm3-Reverse primer	TGACCTTGTCCCCTGCAAAC
Gstm2-Forward primer	CTACCTTGCCCGAAAGCACA
Gstm2-Reverse primer	GCTTCATCTTCTCAGGGAGACC
Gstm4-Forward primer	TGGGTTACTGGGACATCCGT
Gstm4-Reverse primer	TCGGTCATAGTCAGGAGCGT
Sult2a2-Forward primer	CCAAGGGAGATCCGAAGTGG
Sult2a2-Reverse primer	GCCTTGGCCTTGGAACTGAA
Sult2a1-Forward primer	CCAAGGCGATCTATCTCGTG
Sult2a1-Reverse primer	TCCGAATAGAACATTTCCTTTGAG
Sult2a3-Forward primer	TCATTTCCTCTCATCTTCCTGTCC
Sult2a3-Reverse primer	GAGTGATCCTGGATTCTTCACA
Sult2a6-Forward primer	CTCTTCCTGATATGTGGGTTCA
Sult2a6-Reverse primer	GCCAGTTTGTTCCTGACTTGG
Sult1a1-Forward primer	GGTGATCTACGTTGCCCGAA
Sult1a1-Reverse primer	ACCACGACCCATAGGACACT
Sult2a5-Reverse primer	TCCAGTCCCCAGTTGTACCT
Myl2-Forward primer	GGGAGATGCTGACCACACAA
Myl2-Reverse primer	TCCTTCTCTTCTCCGTGGGT
Myh6-Forward primer	TGCTCTCCACCGGGAAAATC
Myh6-Reverse primer	AGAGAATGCGGTTGGGGAAG
Myh7-Forward primer	AGAGGACAGAGGAGCTGGAG
Myh7-Reverse primer	AGCCTGTGCTTGGTCTTCTC
Myl3-Forward primer	CCCAAGGAAGCCGAGTTTGA
Myl3-Reverse primer	ACCTCTGCCTGGGTAGGATT
